# Intake of nutrients (polyunsaturated fatty acids, tocols, and carotenes) and storage efficiency in different slow-growing chickens genotypes reared in extensive systems

**DOI:** 10.1371/journal.pone.0275527

**Published:** 2022-11-01

**Authors:** Simona Mattioli, Alice Cartoni Mancinelli, Alessandro Dal Bosco, Claudia Ciarelli, Monica Guarino Amato, Elisa Angelucci, Diletta Chiattelli, Cesare Castellini

**Affiliations:** 1 Department of Agricultural, Food and Environmental Science, University of Perugia, Perugia, Italy; 2 Department of Agronomy, Food, Natural Resources, Animals and Environment–DAFNAE, University of Padova, Agripolis, Viale dell’Università, Legnaro (PD), Italy; 3 Council for Agricultural Research and Economics, Livestock Production and Aquaculture, Roma, Italy; USDA-Agricultural Research Service, UNITED STATES

## Abstract

An extensive rearing system (ERS) for poultry requires an outdoor run, which enhances the foraging activity of chickens. Slow-growing (SG) strains are more adapted to ERS than fast-growing (FG); and generally, have higher levels of bioactive compounds in their meat. The aim of this paper was to assess the storage efficiency of n-3 and n-6 polyunsaturated fatty acids (PUFA), tocols and carotenes in the meat of seven commercial SG genotypes (SG1-7). One hundred SG chicks/strain of both sexes were included and their walking activity (High- or Low-W) was classified: SG1-4, HW comprised more than 10% of the time budget, and SG5-7, less than 10% (LW). Chickens were reared in pens (4 pens/strain) with indoor (0.10 m^2^/bird) and outdoor (4 m^2^/bird) areas, and they were fed the same diet *ad libitum* (starter feed for 1–21 d, grower feed from 22 d to slaughter at 81 d). The chickens were weighed weekly; feed consumption and grass intake were also estimated. At 81 days of age, 32 chickens/genotype were selected on the basis of the average weight (1:1, M:F) and slaughtered. The breast, thigh and drumstick meat were excised from 30 carcasses/genotype, sampled and stored at -20°C until analysis. Nutrients (e.g., n-3, n-6, carotenes and tocols) of feed, grass and meat were analyzed. The storage efficiency of nutrients was estimated as the ratio between the amount deposited in the body muscles (OUT) and the dietary intake (feed and grass, IN). The genotype affected chickens foraging behavior and the intake of nutrients. For SG1, SG2 and SG3, more than 50% of the intake of n-3 came from grass, whereas in the other genotypes, less than 20%. Accordingly, chickens that foraged more showed better meat nutritional profiles (less fat, more n-3 and antioxidants), which, in ERS, was ascribed to grass ingestion. However, the storage efficiency of nutrients into meat was inversely correlated with the grass intake: strains with higher grass intake (SG1, SG2, and SG3) had lower storage rates. Several hypotheses were proposed to explain these trends.

## Introduction

Extensive rearing systems (ERSs, i.e., organic or free-range rearing) of chicken has received increased interest in Europe. ERSs better meet the welfare and health needs of animals; furthermore, greater consumer interest in more healthy products motivated many chicken companies to develop ERSs [1].

The first EU organic guidelines were provided in 1999 (the implementation of Reg. 2092/91); successively, other rules strictly regulated organic systems for rearing animals (Commission Regulations **2078/1992, 834/2007,** 889/2008, and 848/2018). Adaptation of poultry production to outdoor rearing is essential for complying with the organic guidelines. The chicken density in ERSs is 28 kg/m^2^ (compared to 21 kg/m^2^ in organic EU systems); ERSs require access to a pasture (4 m^2^/chicken) and environmental enrichment [2,3] that enhance poultry activity. In the pasture, chickens exhibit the range of their behavioral repertoire (e.g., preening, walking and foraging; [4–6]) while exploring the outdoor area and eating grass, worms and insects. Chickens adapted to this natural rearing environment generally have better immunity, higher resistance to diseases, and high-quality meat [7–9] due to the intake of bioactive compounds (Poly-Unsaturated Fatty Acids-PUFA, vitamins, and carotenes [10]).

The genetic strain modulates the foraging activity of chickens: chickens with high growth rates (mainly selected for intensive rearing systems) generally have low locomotor activity and are not adapted to organic systems [11]. Accordingly, EU Regulation 848/2018 recommends the use of slow-growing strains for organic systems and introduces the concept of adaptability to outdoor rearing. Mancinelli et al. [6] demonstrated that although a prerequisite of adaptability to ERS is a daily weight gain (DWG) below 45 g/d, the genotype can also affect this adaptability. Accordingly, slow weight gain is a prerequisite of adaptation, but SG chickens also differ in other aspects, such as walking and foraging behavior [8,9,12,13], demonstrating that interest in outdoor spaces strongly depends on the intrinsic behavior and genetics of animals.

However, because access to a pasture is mandatory in ERSs, the grass intake and storage ability of nutrients originating from grass by different strains has attracted increasing attention. Studies on the storage efficiency of nutrients in ERSs are scarce [14,15] and require the development of new methodological tools. In aquaculture, feed storage efficiency is determined as the amount of intake with respect to the amount stored in the body (the Fish In/Fish Out index [16]). In the light of what reported, the objective of the present research was to estimate the intake of some nutrients (n-3 and n-6 PUFA, tocols and carotenes) and their body (muscle) storage in different commercial SG poultry genotypes.

## Material and methods

### Animals and farming system

The trial was carried out at the experimental farm of the University of Perugia (Italy) in September-November 2020. Chickens were reared according to EU Regulation 834/07 and 889/2008, and the Italian directives [17] on animal welfare for experimental and other scientific purposes. The experimental protocol was approved by the Ethical Committee of the University of Perugia (ID number: 62705 of 07/15/2020).

A total of 100 chickens/genotype (25 chickens x 4 replicates) of both sexes (male:female ratio of 1:1) from seven SG genotypes were used. The birds were provided by two commercial poultry farms: three strains from Aviagen (Ranger Classic–SG7, Ranger Gold–SG4, Rowan Ranger–SG1, RedJA–SG3; Cocconato, AT, Italy) and four strains from Hubbard (CY Gen 5 JA87 –SG6, M22 × JA87 –SG5, Naked Neck–SG2; Le Foeil-Quintin, France), and selected on the basis of different growth rates (DWG, g/d/bird, Table 1) and live weights (Table 1). According to previous studies [6,9], the genetic lines were classified on the basis of walking behavior (SG1-4 spent > 10% of budget time in walking activity, whereas SG5-7 spent ≤ 10% of budget time in walking activity).

**Table 1:** Genetic lines studied, daily weight gain (DWG, g/d/bird), live weight (g ± SEM) and walking behaviour.

Acronym ^a^	DWG ^b^, g/d/bird	live weight, g	walking behaviours ^c^
SG1	32.11	3957.14±318.07	HW
SG2	32.50	3682.14±542.91	HW
SG3	34.91	3405.00±202.01	HW
SG4	41.90	3363.57±228.23	HW
SG5	42.36	2579.28±356.49	LW
SG6	44.83	2783.57±273.51	LW
SG7	48.47	2632.28±127.48	LW

^a^ SG1: Rowan Ranger, SG2: Naked Neck, SG3: RedJA, SG4: Ranger Gold, SG5: M22 × JA87, SG6: CY Gen 5 JA87, SG7: Ranger Classic.

^b^ DWG: Daily weight gain by Cartoni Mancinelli et al. [5].

^c^ HW: High-Walking, NW: Low-Walking by Pulcini et al. [9].

Each chicken strain was reared in four different pens that provided 128 m^2^ of outdoor space/replicate (total pen dimensions: 32 x 32 m) and which were also equipped with a shelter. The indoor (0.10 m^2^/bird) and outdoor (4 m^2^/bird) densities of animals were specified according to organic regulations (EC Regulation nos. 834/2007 and 889/2008). From 1 to 20 days of age, birds were housed in an environmentally controlled poultry house, with a temperature between 30 and 32°C and relative humidity oscillating between 65% and 70%. At 21 days of age, the chickens were provided with free access to the outdoor space. The temperature and humidity of the pasture were 19±7°C and 50.1±12.5%, respectively. The pasture was not treated with pesticides and contained natural bushes and hedges (Table 2).

**Table 2:** Floristic composition of pasture.

*Phleum sp*.	*Avena fatua*
*Dactylis glomerata*	*Sanguisorba minor*
*Santolina sp*.	*Linaria sp*.
*Agropyron sp*.	*Picris hieracioides*
*Calamintha nepeta*	*Reichardia picroides*
*Rubus sp*.	*Daucus carota*
*Chondrilla juncea*	*Geranium sp*.
*Cichorium intybus*	*Euphorbia sp*.
*Centaurea sp*.	*Campanula rapunculus*
*Convolvulus sp*.	*Portulaca oleracea*
*Plantago lanceolata*	*Petrorhagia prolifera*

The animals were fed *ad libitum* with the same diet (starter feed for 1–21 d, grower feed from 22 d to slaughter; Table 3); the diets provided chicken nutritional requirements as recommended by the breeding companies and scientific literature [18]. Water was always available, and the birds were kept in shelters only during the night to protect them from predators.

**Table 3:** Dietary ingredients, proximate composition, energy value and nutrients of feed and grass.

		Starter	Finisher	Grass
*Ingredients*				
Maize	%	53.92	53.11	
Soybean meal	“	30.23	15.69	
Wheat	“	5.00	15.00	
Maize meal	“	5.08	11.45	
Gluten feed	“	1.00		
Soybean oil	“	0.62	1.15	
Vitamin-mineral premix ^a^	“	0.40	0.40	
Dicalcium phosphate	“	1.71	1.21	
Calcium carbonate	“	1.23	1.29	
NaCl	“	0.20	0.23	
Sodium bicarbonate	“	0.15	0.15	
*Proximate composition*				
Moisture	%	12.20	12.00	78.61
Crude protein	% of DM	24.01	18.41	8.34
Ether extract	“	3.99	4.55	2.11
Ash	“	6.92	5.78	7.85
Crude fibre	“	3.48	3.60	23.2
NDF	“	17.63	10.1	60.90
ADF	“	7.41	5.06	39.81
ADL	“	1.67	1.11	5.81
Cellulose	“	5.74	3.56	34.0
Hemicellulose	“	10.22	5.05	21.09
Metabolizable energy ^b^	kcal/kg	3245.20	3295.94	1876.00
*Nutrients* ^*c*^				
Vitamin A	mg/kg of D.M.	14.3	14.55	-
Vitamin E	“	67.5	55.03	355.51
Carotenes	“	2.16	3.65	401.65
C16:0	g/kg of D.M.	0.84	0.86	5.00
C16:1	“	0.01	0.01	0.21
C18:0	“	0.15	0.20	1.06
C18:1	“	1.60	1.65	7.53
C18:2	“	3.52	3.58	8.16
C18:3	“	0.27	0.29	8.56
SFA	“	0.99	1.06	6.05
MUFA	“	1.61	1.66	7.74
PUFA	“	3.79	3.87	16.72
n-6	“	3.52	3.58	8.16
n-3	“	0.27	0.29	8.56
n-6/n-3	-	13.04	12.34	0.95

^a^ Amount per kg: vitamin A, 11,000 IU; vitamin D_3_, 2000 IU; vitamin B_1_, 2.5 mg; vitamin B_2_, 4 mg; vitamin B_6_, 1.25 mg; vitamin B_12_, 0.01 mg; α-tocopheryl acetate, 30 mg; biotin, 0.06 mg; vitamin K, 2.5 mg; niacin, 15 mg; folic acid, 0.30 mg; pantothenic acid, 10 mg; choline chloride, 600 mg; manganese, 60 mg; iron, 50 mg; zinc, 15 mg; iodine, 0.5 mg; and cobalt, 0.5 mg.

^b^ Estimated by Carrè and Rozo [19].

^c^ SFA: Saturated fatty acid, MUFA: Monounsaturated fatty acid; PUFA: Polyunsaturated fatty acid.

Once a week, 25 chickens/replicate from all genotypes were weighed. The feed consumption was recorded in every replicate by weighing the quantity of feed provided minus the feed that remained at the end of the week according to weight. The feed intake referred to intake over 80 days (from 1 to 81 days).

### Grass intake

The modified method of Lantinga et al. [20] was applied to estimate forage intake. At the start of the rearing cycle, a metallic frame (exclusion pens, 0.50 × 0.50 m) was positioned 10 m from the shelters in each replication. Herbage samples were collected at the beginning (outside the exclusion pens) and at the end (both inside and outside the exclusion pens) of the rearing cycle in each replication. Outside the exclusion pens, collections were carried out from one area of the same size (i.e., 0.50 × 0.50 m) randomly chosen by casting a frame on the ground, approximately one meter away from each exclusion pen, to provide the same number of observations for grazed and undisturbed areas.

Grass intake (GI) was estimated using the following equation:

GI=(GMs‐GMe)+[[1‐(GMe/GMs)/‐In[GMe/GMs]]x(GMu‐GMs)]

where GMs = herbage present before birds entered each pen; GMe = forage that remained at the end of the trial; and GMu = undisturbed forage mass from the exclusion pens.

The forage intake was representative of the whole subarea of the pens. The values obtained from the different pens were then averaged and referred to 59 days of outdoor access (from 21 to 80 days).

### Production performance and carcass traits

At 81 days of age, 32 chickens/genotype were selected on the basis of the average weight (± 10%) and slaughtered (8 chickens/replicates/genotypes, M:F 1:1) in a commercial slaughterhouse 12 h after feed withdrawal. The animals were electrically stunned (110 V; 350 Hz) before being killed. After bleeding, the carcasses were placed in hot water (56.5°C for 1 min) and then plucked and eviscerated (nonedible viscera, including intestines, proventriculus, gall bladder, spleen, esophagus, and full crop were removed), and the carcasses were stored for 24 h at 4°C. The breast, thigh and drumstick muscle were excised from 30 carcasses/genotypes, separately sampled and stored at -20°C, until the analysis.

### Proximate composition of feed and grass

Moisture was determined by oven-drying at 105°C overnight an aliquot of feed and grass [21]. Crude protein was measured by a Kjeldahl nitrogen analysis [21]. Ether extract were quantified by diethyl ether using a Soxhlet apparatus (SER 148, VELP Scientifica, Monza-Brianza, Italy). Ash content was determined by combusting for 3 h at 550°C. Crude fiber was determined as described by Reference [21]. Crude fiber, neutral detergent fiber (NDF), acid detergent fiber (ADF), and acid detergent lignin (ADL) content were determined according to Reference [22]. Cellulose and hemicellulose were calculated as differences started from NDF, ADF and ADL (i.e., cellulose = ADF-ADL; hemicellulose = NDF-ADF). Metabolizable energy were estimated following what reported by Carrè and Rozo [19].

### Nutrients in feed, grass and meat

Nutrients in feed, grass and meat were analyzed in duplicate, furthermore the different meat cuts were analysed separately.

#### Antioxidants

The α-, γ-, and δ-tocopherol; α- and γ-tocotrienol; carotenes (feed and grass) and retinol (meat) levels were quantified using HPLC (Hitachi Primade, Milan, Italy) according to Hewavitharana et al. [23]. Five milliliters of distilled water and 4 mL of ethanol were added to 2 g of sample and vortexed for 10 s. After mixing, 4 mL of hexane containing BHT (200 mg/L) was added, and the mixture was carefully shaken and centrifuged at 8,000 × g for 10 min. An aliquot of the supernatant (3 mL) was dried under a stream of nitrogen and dissolved in 200 μL of acetonitrile; 50 μL was then injected into the same HPLC system (Hitachi Primade comprised of a cooling autosampler 1210, pump 1110, fluorimetric detector 1440 and diode array detector 1430 and a Synergi Hydro-RP column, Phenomenx, Bologna, Italy). The antioxidant content in feed, grass and meat were expressed as mg/kg. The average amount of each nutrients was used to calculate the daily intake (μg/d). The sum of tocols (tocotrienols + tocopherols) and carotenes (lutein + zeaxanthin) intake in grass and feed was also calculated.

#### Fatty acids

Fatty acid intake was evaluated from the lipid fraction extracted from feed, grass and meat following the method reported by Folch et al. [24]. To measure the fatty acid methyl esters, the lipid extract was dried with a rotavapor, and 1 mL of n-exane was added. Finally, the trans-metylation procedure was performed with 0.5 mL of 2 M KOH methanol solution at 60°C for 15 min. One microliter was added to the gas chromatography system (CP 3800 VARIAN, Milan, Italy) equipped with an FID detector and a capillary column of 100 m length x 0.25 mm x 0.2 μm film (Supelco, Bellefonte, PA). To calculate the amount of each fatty acid, heneicosanoic acid was used as the internal standard (C21:0, Sigma–Aldrich analytical standard). The amount of each fatty acid was expressed as mg/100g of tissue and used to calculate the total saturated (SFA), total monounsaturated fatty acids (MUFA), and total PUFA from the n-3 and n-6 series.

### Estimation of storage efficiency: OUT/IN ratio

We modified the in/out index [16] into the opposite (out/in) to directly assess the storage efficiency of dietary sources in body muscle. This index was developed for the different dietary sources (feed and grass) and for the specific nutrients measured. Thus, the storage efficiencies were estimated using a simple OUT/IN ratio, not taking into account all the metabolic (catabolic) mechanisms that can be modified it [25].

After estimating the feed and grass intake (see previous sections) of the seven chicken strains, the intake of each nutrients (n-3 and n-6 PUFA, tocols and carotenes) was calculated (mg). Then, we calculated the OUT/IN ratio for the entire length of the experiment using the equation:

OUT/IN = (compounds on breast, thing and drumstick) / [(daily intake of compounds by grass X 59) + (daily intake of compounds by feed X 80)]

- The sum of breast, thing and drumstick meat was used because represents more than 60% of chicken muscle mass and more than 80% of commercial meat cuts [26];
- The animals had access to the outdoors for 59 days;
- The entire rearing period (chickens life) lasted 80 days (81 days minus 1 day because the day before slaughtering, food was withheld).

### Statistical analysis

**Fig 1:**
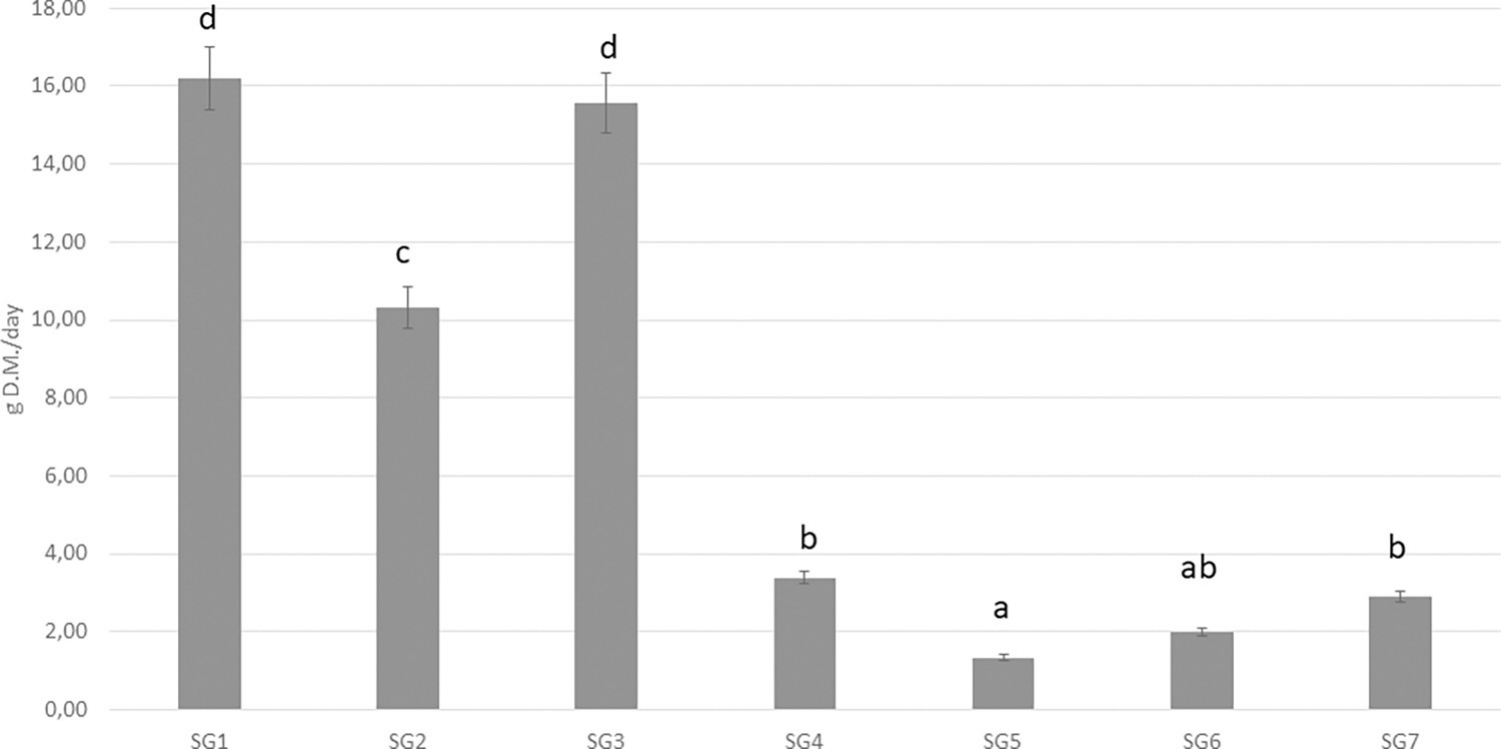
Estimated grass intake of different chicken genotypes along the trial. Grass intake is expressed as g D.M./day and presented as mean ± SE. a..d means P < 0.01. SG1: Rowan Ranger, SG2: Naked Neck, SG3: RedJA, SG4: Ranger Gold, SG5: M22 × JA87, SG6: CY Gen 5 JA87, SG7: Ranger Classic. SG1, SG2, SG3, SG4: High-Walking chickens; SG5, SG6, SG7: Low-Walking chickens.

**Fig 2:**
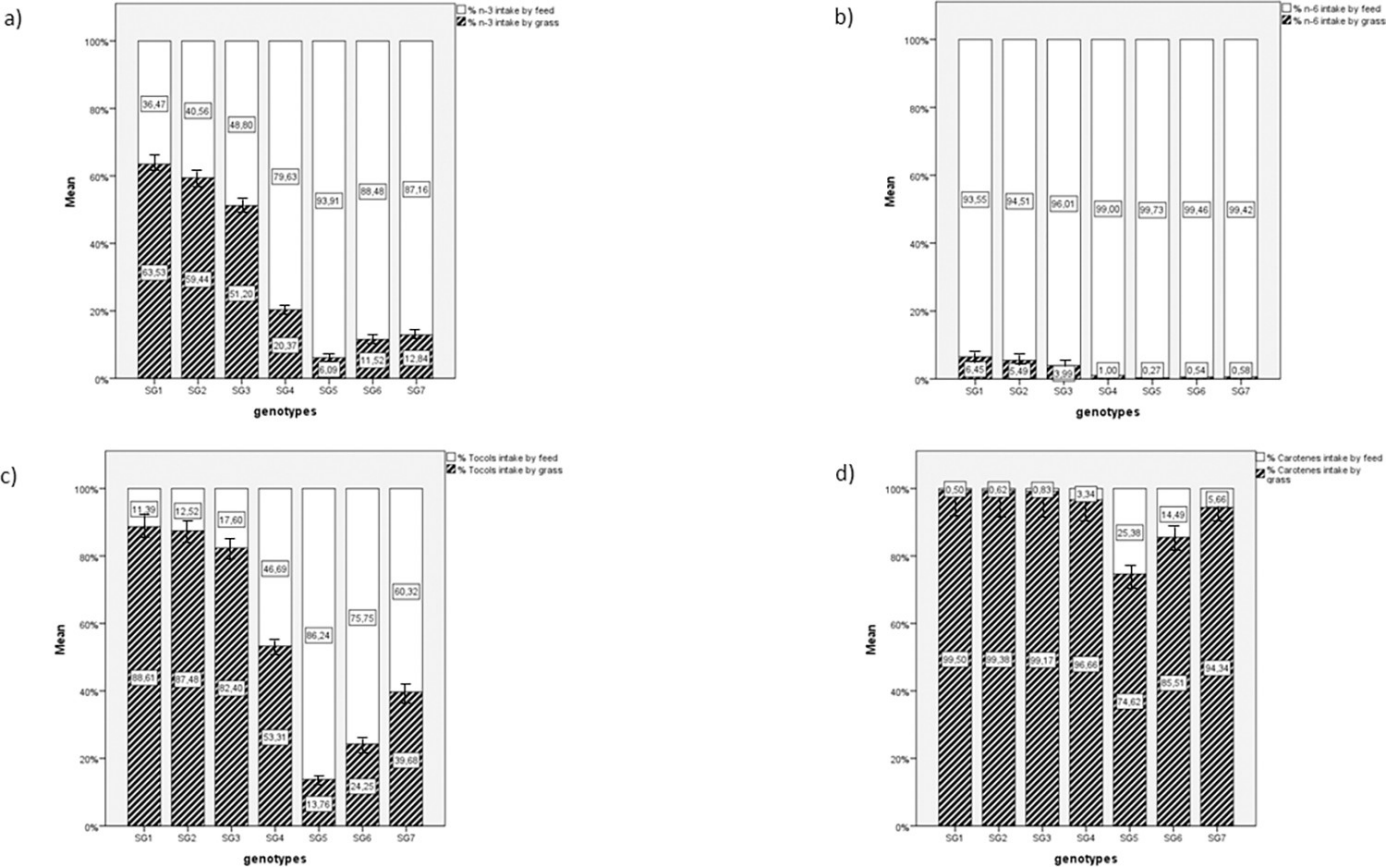
Proportion of n-3 PUFA, n-6 PUFA, tocols and carotenoids furnished by feed and grass intake. The n-3 PUFA (a), n-6 PUFA (b), tocols (c) and carotenoids (d) are expressed as %. White bar represents intakes by feed. Black bar represents intakes by grass. SG1: Rowan Ranger, SG2: Naked Neck, SG3: RedJA, SG4: Ranger Gold, SG5: M22 × JA87, SG6: CY Gen 5 JA87, SG7: Ranger Classic. SG1, SG2, SG3, SG4: High-Walking chickens; SG5, SG6, SG7: Low-Walking chickens. Upper and lower limits was set at 95%.

**Fig 3:**
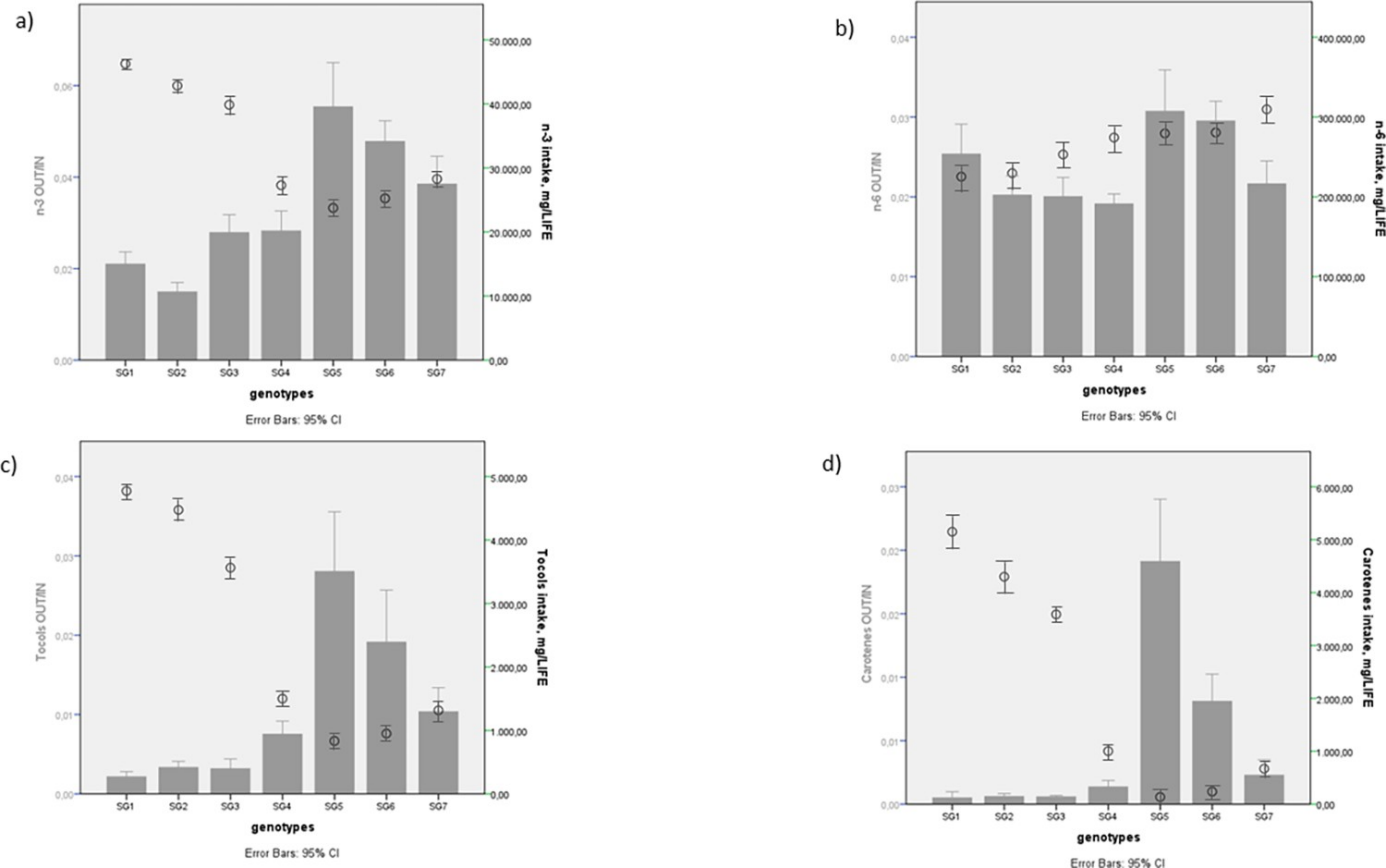
Storage efficiency into chicken body and n-3 PUFA, n-6 PUFA, tocols and carotenes intake. Grey full bar represents the storage efficiency expressed as OUT/IN ratio; black empty dot represents n-3 PUFA (a), n-6 PUFA (b), tocols (c) and carotenes (d) intakes expressed as mg/life. SG1: Rowan Ranger, SG2: Naked Neck, SG3: RedJA, SG4: Ranger Gold, SG5: M22 × JA87, SG6: CY Gen 5 JA87, SG7: Ranger Classic. SG1, SG2, SG3, SG4: High-Walking chickens; SG5, SG6, SG7: Low-Walking chickens. Upper and lower limits 95% of confidence.

**Fig 4:**
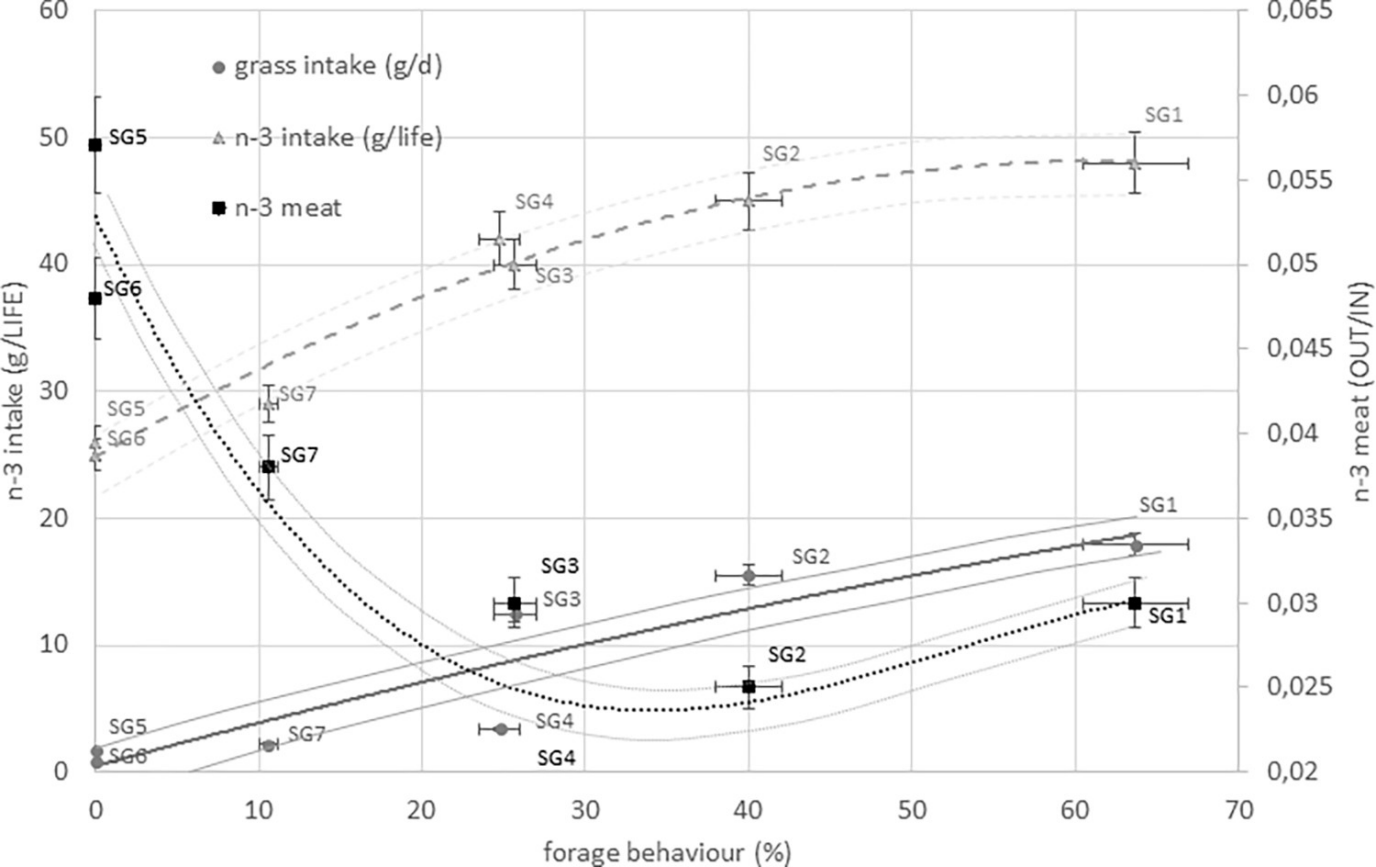
Grass and n-3 intakes and storage efficiency, in relation to the foraging behaviors of chicken genotypes. Dark gray dot and solid line represent respectively the grass intake and n-3 intake expressed as g/d; light gray triangle and dotted line represents n-3 intake expressed as g/life; black square and solid line represent storage efficiency expressed as OUT/IN ratio; foraging behaviors is expressed as %. SG1: Rowan Ranger, SG2: Naked Neck, SG3: RedJA, SG4: Ranger Gold, SG5: M22 × JA87, SG6: CY Gen 5 JA87, SG7: Ranger Classic. SG1, SG2, SG3, SG4: High-Walking chickens; SG5, SG6, SG7: Low-Walking chickens.

Linear models (SPSS v. 27, Italy) were used to evaluate the effect of chicken genotype. For the feed/grass intake the pens (n = 4/genotype) have been considered as experimental units, whereas for the chickens live weight and quantification of nutrients in meat cuts the individual carcasses were considered (n = 30/genotype). Differences among strains were assessed by one-way ANOVA with Tukey’s test for multiple comparisons. Differences with a P < 0.05 were considered statistically significant. The graphs were constructed in Microsoft Office Excel (Figs 1 and 4) and SPSS software (Figs 2 and 3), and the data were expressed as the means and 95% confidence intervals. Polynomial regressions were fitted to show the grass and n-3 fatty acids intake and the OUT/IN trend in relation to chicken foraging behavior. Upper and lower 95% confidence limits were also reported.

## Results

Table 3 reports the feed ingredients and the chemical composition of the grass and feed. The grass had a high water content; however, if comparisons were conducted with DM, the bioactive compound content in grass was much higher than that in feed (e.g., vitamin E, carotenes, n-3 PUFA, 7, 120 and 40 times, respectively).

Fig 1 shows the grass intake of the chicken genotypes. SG1 ate the most grass (16.12 g of DM/d), followed by SG3 and SG2 (15.68 and 10.52 g of DM/d, respectively), while SG4, SG7, SG6 and SG5 ate little grass quantities (3.39, 2.22, 1.70, 0.85 g of DM/d, respectively).

In Tables 4 and 5, the ingestion of nutrients ascribed to grass and feed, respectively, are reported. Grass (Table 4) provided higher levels of antioxidants (mainly carotenes: lutein + zeaxanthin) and n-3 PUFA than those of feed, corresponding to the grass intake of different strains.

**Table 4:** Estimated nutrients intake through grass.

	Genotype ^a^								
	SG1	SG2	SG3	SG4	SG5	SG6	SG7	RMSE ^b^	P value
*Antioxidants*									
α-Tocotrienol	8.59d	6.02c	5.96c	1.63b	0.31a	0.63a	1.06b	0.66	<0.001
δ-Tocopherol	1.90	1.65	1.32	0.36	0.16	0.32	0.24	0.31	0.126
γ-Tocopherol	0.70	0.65	0.49	0.13	0.03	0.05	0.09	0.19	0.305
α-Tocopherol	60.46c	58.04c	41.99c	11.45b	1.45a	2.91a	7.50b	1.87	<0.001
Lutein + Zeaxanthin	86.81d	72.45c	60.28c	16.44b	1.72a	3.44a	10.77b	2.19	<0.001
*Fatty acids* ^c^									
C16	162.82d	141.17d	113.07d	30.84c	8.76a	17.52b	20.19b	6.97	<0.001
C16:1	11.17c	9.68c	7.75c	2.11b	0.16a	0.31a	1.38b	1.42	<0.001
C18	46.35d	40.19d	32.18d	8.78c	2.06a	4.11b	5.75b	3.20	<0.001
C18:1 n-9	628.01e	544.51e	436.10e	118.94d	27.43a	54.87b	77.89c	8.56	<0.001
C18:2 n-6, LA	246.41e	213.64e	171.11d	46.67c	12.93a	25.85b	30.56b	8.91	<0.001
C18:3 n-3, α-ALA	498.09e	431.86e	345.88d	94.33c	24.65a	49.30b	61.77b	9.13	<0.001
SFA	209.17d	181.36d	145.25d	39.61c	10.82a	21.63b	25.94b	7.68	<0.001
MUFA	639.18e	554.19e	443.85e	121.05d	27.59a	55.18b	79.27c	8.68	<0.001
PUFA	744.49f	645.51e	516.99e	141.00d	37.58a	75.16b	92.33c	12.76	<0.001

Grass nutrients intake is expressed as mg/d.

a..f, P < 0.001.

^a^ SG1: Rowan Ranger, SG2: Naked Neck, SG3: RedJA, SG4: Ranger Gold, SG5: M22 × JA87, SG6: CY Gen 5 JA87, SG7: Ranger Classic. SG1, SG2, SG3, SG4: High-Walking chickens; SG5, SG6, SG7: Low-Walking chickens.

^b^ RMSE: Root mean square error.

^c^ LA: Linoleic Acid, ALA: Linolenic Acid, SFA: Saturated Fatty Acid, MUFA: Mono Unsaturated Fatty Acid; PUFA: Poly-Unsaturated Fatty Acid.

**Table 5:** Estimated nutrients intake through feed.

	Genotype ^a^									
	SG1	SG2	SG3	SG4	SG5	SG6	SG7	RMSE ^b^		P value
*Antioxidants*										
α-Tocotrienol	2.84a	2.93a	3.28b	3.67b	3.76bc	3.76bc	4.16c	0.27		<0.001
δ-Tocopherol	0.67	0.69	0.77	0.86	0.88	0.89	0.98	0.13		0.406
γ-Tocopherol	0.22	0.23	0.25	0.28	0.29	0.29	0.32	0.07		0.268
α-Tocopherol	2.97a	3.07a	3.43b	3.84b	3.94bc	3.94bc	4.36c	0.27		<0.001
Lutein + Zeaxanthin	0.32	0.33	0.37	0.41	0.42	0.42	0.47	0.05		0.103
*Fatty acids* ^c^										
C16	622.13	641.08	718.01	803.03	823.38	824.43	911.75	11.55		0.223
C16:1	4.76	4.91	5.49	6.15	6.30	6.31	6.98	0.15		0.804
C18	154.01	158.70	177.74	198.79	203.83	204.09	225.71	8.71		0.099
C18:1 n-9	1243.09	1280.95	1434.66	1604.55	1645.20	1647.30	1821.79	22.13		0.126
C18:2 n-6, LA	2598.21a	2677.33a	2998.61b	3353.71b	3438.67b	3443.05b	3807.76c	37.17		<0.001
C18:3 n-3, ALA	208.01a	214.35a	240.07b	268.50b	275.30bc	275.65bc	304.85d	12.89		<0.001
SFA	776.14	799.78	895.75	1001.83	1027.21	1028.52	1137.46	21.71		0.340
MUFA	1247.85a	1285.85a	1440.15b	1610.70c	1651.51c	1653.61c	1828.77d	29.14		<0.001
PUFA	2806.22a	2891.67a	3238.67b	3622.20b	3713.97b	3718.70b	4112.60c	12.30		<0.001

Feed nutrients intake is expressed as mg/d.

a..d, P < 0.001.

^a^ SG1: Rowan Ranger, SG2: Naked Neck, SG3: RedJA, SG4: Ranger Gold, SG5: M22 × JA87, SG6: CY Gen 5 JA87, SG7: Ranger Classic. SG1, SG2, SG3, SG4: High-Walking chickens; SG5, SG6, SG7: Low-Walking chickens.

^b^ RMSE: Root mean square error.

^c^ LA: Linoleic Acid, ALA: Linolenic Acid, SFA: Saturated Fatty Acid, MUFA: Mono Unsaturated Fatty Acid; PUFA: Poly-Unsaturated Fatty Acid.

Similarly, the contribution of feed (Table 5) was different, related to the genetic strains; however, the differences were lower than those of grass.

Consequently, the proportion of n-3 and n-6 PUFA, tocols and carotenes provided by feed and grass was widely affected by the foraging preferences of the genotypes (Fig 2). For SG1, SG2 and SG3, more than 50% of the intake of n-3 PUFA came from grass; in the other genotypes, less than 2% of this intake came from grass (Fig 2A). Conversely, n-6 PUFA were mainly furnished by feed, and in each genotype, feed provided more than 93% of n-6 PUFA (Fig 2B).

The antioxidant intake (carotenes and tocols; Fig 2C and 2D) was also modulated by grass ingestion, with SG1, SG2, and SG3 having higher values. SG6 and SG5 ingested 75.73 and 66.17% of tocols, respectively, from feed; carotenes were almost entirely provided by grass in all genotypes.

The storage efficiency (OUT/IN ratio) of nutrients in the body differed (Fig 3A–3D). The mean body storage varied by compound (from approximately 1 for tocols to 2.5 and 3.5 for n-6 and n-3 PUFA, respectively) and by genetic strain.

Generally, the chickens with higher grass intake (SG1 and SG2) had lower storage efficiency of n-3 PUFA and tocols, and thus a negative correlation with the grass intake (P<0.01; Table 6).

**Table 6:** Correlation between grass intake and storage efficiency of n-3, n-6 PUFA, tocols and carotenes.

|	Grass intake	n-3 PUFA ^a^	n-6 PUFA ^a^	Tocols
n-3 PUFA ^a^	-0.83**	-	-	-
n-6 PUFA ^a^	-0.31*	0.78**	-	-
Tocols	-0.77**	0.96**	0.82**	-
Carotenes	-0.58**	0.79**	0.70**	0.88**

Grass intake is expressed as g D.M./day; storage efficiency is expressed as OUT/IN ratio.

*correlation is significant at the 0.05 level.

**correlation is significant at the 0.01 level.

^a^ PUFA: Poly-Unsaturated Fatty Acid.

The trend of n-6 PUFA storage was different than that of n-3 PUFA; SG6 and SG5 showed significantly different levels (in average 1.5 times higher) than those of the other genotypes (Fig 3B).

SG1, SG2 and SG3 presented the lowest tocols OUT/IN ratio, followed by SG4 and SG7; SG5 and SG6 exhibited the highest OUT/IN ratio (Fig 3C). Carotene metabolism (Fig 3D), estimated as meat retinol concentration resulting from lutein and zeaxanthin intake, followed the same trend as that of tocols.

The fatty acid profile (Table 7) of chicken meat showed that strains with higher grass intake (i.e., SG1 and SG3), had n-3 fatty acid with more than 20 carbon atoms (long-chain polyunsaturated fatty acids, LC-PUFA) levels similar to those of SG6, a strain that exhibited less foraging behavior. Accordingly, grass intake could be a useful way to increase LC-PUFA content in meat, magnifying the low ALA content of standard feed, although the conversion efficiency of the n-3 precursor (C18:3n-3, ALA) into n-3 LC-PUFA decreased with grass ingestion.

**Table 7:** Fat and main PUFA content of body chicken meat.

	Genotype ^a^								
	SG1	SG2	SG3	SG4	SG5	SG6	SG7	RMSE ^b^	P value
Fat	2.60b	1.78a	2.38b	1.90a	2.49b	2.97c	1.90a	0.24	<0.001
*Fatty acids* ^c^									
C18:2n-6, LA	460.94b	389.08a	377.96a	346.22a	449.08b	496.87b	343.22a	2.88	<0.001
C18:3 n-3, ALA	51.88c	27.2a	59.15c	37.03a	46.90b	52.21c	37.99a	1.27	<0.001
C18:4 n-3	0.97a	1.65a	1.53a	2.13b	2.80c	2.00b	2.09b	0.36	0.01
C20:3 n-3	0.95b	0.64a	0.64a	0.70ab	0.86b	1.20c	0.52a	0.20	<0.001
C20:4 n-6 AA	120.65c	87.17a	115.55b	109.80b	127.03c	185.5d	98.96b	2.12	<0.001
C20:5 n-3, EPA	4.75b	15.07c	3.35b	2.57a	1.95a	2.60a	2.21a	0.84	<0.001
C22:5 n-3, DPA	22.45b	11.91a	27.36c	14.10a	27.40c	25.80bc	19.93b	0.99	<0.001
C22:6 n-3, DHA	19.86c	13.55b	18.55c	11.08b	9.01a	13.00b	9.67a	0.80	<0.001
LC-PUFA n-3	47.04c	39.52b	48.37c	26.32a	36.96b	41.60bc	30.24a	0.20	<0.001

Fat is expressed as %; main PUFA are expressed as mg/100 g of meat.

a..c P < 0.01.

^a^ SG1: Rowan Ranger, SG2: Naked Neck, SG3: RedJA, SG4: Ranger Gold, SG5: M22 × JA87, SG6: CY Gen 5 JA87, SG7: Ranger Classic. SG1, SG2, SG3, SG4: High-Walking chickens; SG5, SG6, SG7: Low-Walking chickens.

^b^ RMSE: Root mean square error. ^c^ LA: Linoleic Acid, ALA: Linolenic Acid, SFA: Saturated Fatty Acid, MUFA: Mono Unsaturated ^c^ Fatty Acid; PUFA: Poly-Unsaturated Fatty Acid, LC-PUFA: Long-Chain PUFA.

Fig 4 shows the n-3 PUFA storage ability of chickens on the basis of their grass intake. Higher conversion efficiencies were found in strains that foraged more (mainly in the SG1, SG2 and SG3 genotypes), primarily ascribed to the n-3 PUFA intake furnished by grass.

## Discussion

In well-managed ERSs, the foraging activity of chickens may replace up to 5–20% of feed [2,27]. However, this replacement depends on several factors that determine how appealing a plant is to a bird, such as the plant species, nutritional content, and stage of growth, as well as individual bird, such as its nutritional needs, hunger, and foraging and feeding behavior [28,29]. Almeida et al. [30] found that protein-restricted diets motivated grass ingestion by chickens. However, other studies and the experience of poultry producers suggest that chickens consume large amounts of forage even if they have good feed available: poultry crave greens and eat them even if balanced feed is provided [31].

In our study, the grass intake was moderate in all chickens (from 0.85 to 17.90 g of DM/d), but different grass intakes modified the proportion of some nutrients (n-3 and n-6 PUFA, tocols and carotenes) ingested by poultry genotypes. The SG1 birds had the highest intake of pasture (17.90 g of DM/day), whereas the SG5 birds had the lowest. SG genotypes are more active than FG [32], and the negative correlation between foraging behavior and productive performance is well documented [8].

Previous trials have confirmed that feeding behaviors vary among poultry strains. Lorenz et al. [33] found that slow-growing chickens had higher grass intake than fast-growing chickens. Castellini et al. [34] determined that the crop content of “scavenger” chickens, compared to that of fast-growing strains, had less protein and energy and higher amounts of α-tocopherol and carotenes, indicating greater grass ingestion. Conversely, breast meat from broiler chickens with free access to pasture presented lower levels of n-6 and n-3 fatty acid precursors compared to SG chickens, which increased during the spring season, suggesting that storage efficiency varies depending on the type of pasture available [35].

Genetic selection has deeply modified the behavior of chickens, and SG strains show different foraging behaviors (i.e. grass intake) [36]. Pasture-raised birds still require a grain-based feed formulated for sustaining body growth. Moreover, with very high grass intake, the fiber content may limit the digestibility of nutrients and feed efficiency. In our previous study, the circulating levels of antioxidants (mainly vitamin E) in blood [8] suggest a minor effect of grass fiber, which were almost independent of grass intake, although the effect of grass ingestion on digestibility cannot be excluded. However, data on the amounts and types of nutrients foraged by free-range birds are lacking and should be further scrutinized to formulate diets that can maintain high poultry production and meat quality. Furthermore, it should be emphasized that free-range birds also consume roots, stems and invertebrates [37].

Access to vegetation provides a multitude of vitamins, minerals and lipids. Dal Bosco et al. [10] showed that vitamins, minerals and n-3 PUFA are transferred from vegetation to the meat of organic chickens, also affecting the oxidative stability of the meat (increase antioxidants content, reduce lipid oxidation). In particular, the PUFA profile of grass differs from that of feed (e.g., n-6/n-3 0.90 vs. 13.04 and 12.34; Table 3) and represents a way to increase n-3 PUFA intake in poultry diets. Increasing grass intake also increases the n-3 PUFA content in meat and eggs [32,38–41].

The present results indicate that the foraging profile of a genotype largely affects the availability of nutrients because chicken genotypes respond differently to the presence of an outdoor range [5,8,9], in terms of both walking activity and forage intake. The proportion of n-3 and n-6 PUFA, tocols and carotenes in the examined strains were affected by their relative intake of grass (Table 4) and feed (Table 5, Fig 2). More than 50% of the n-3 PUFA intake of the “foraging” strains (SG1, SG2 and SG3) was provided by grass, while in the other genotypes, grass provided less than 20%. Therefore, in the former genotypes, n-3 PUFA intake strongly depends on pasture. Conversely, n-6 PUFA were mainly furnished by feed (> 93%). Indeed, the common ingredients of poultry feed (e.g., corn and soybeans) have fatty acid profiles mainly consisting of n-6 PUFA [42,43].

Furthermore, grass represented an important source of carotenes for all chicken genotypes (Table 4; Fig 2). These compounds mainly act as antioxidants or provitamins (i.e., β-carotene [44]). More than 1,100 carotenes have been identified in vegetation, including xanthophylls, β-cryptoxanthin, astaxanthin, canthaxanthin, zeaxanthin and lutein, but many of these do not have a provitamin effect [45]. Furthermore, zeaxanthin and xanthophylls (derived from corn and alfalfa, respectively), are commonly found in poultry feeds and contribute to skin yellowing and the yellow/orange color of egg yolks [46]. In addition, carotenes have also been implicated in the modulation of the innate immune system [47,48].

The intake of tocols and the proportion furnished by feed and grass (Fig 3C) also varied by genotype and were higher in SG1, SG2, and SG3. In SG6 and SG5, the intake of tocols was mainly furnished by feed. Indeed, tocopherol-acetate is generally added to the poultry diets to improve the shelf life of meat, [49], greatly contributing to the tocols furnished by feed.

The present study estimated the specific intake of different compounds furnished by different sources (grass and feed) of chicken strains to assess the relative storage efficiency in body muscle (OUT/IN ratio). Naturally, the other body tissues and organs could play a key role in storage (i.e. liver and fat); while, other tissues (many organs) are thought to have a minor role [50] and mainly preserve normal physiological functions, including immunity, health, and homeostasis.

The nutrients storage efficiency in body meat of chicken strains (Fig 3) differed from a ratio of approximately 1 for tocols (Fig 3C) to approximately 6 for n-3 PUFA (Fig 3A). The chicken strains with higher grass intake (SG2, SG1, and to a lesser extent SG3) had lower storage efficiency, indicating that as grass intake increases, storage ability decreases (Fig 4). The genotypes with the highest storage efficiency were SG5 and SG6, although they had a low intake.

Many authors have reported a higher percentage of n-3 PUFA (g n-3 PUFA/100 g fatty acids) in the breast and drumstick meat of more active poultry genotypes [10,14,51]. Our data are consistent with the above-mentioned results (Table 7). However, our results indicated that genotypes with higher DWG, such as SG5 and SG6, also have a higher storage efficiency, probably because the content of fatty acids is widely modulated by the lipid content and then by their amount in meat. Indeed, these strains have meat with higher fat content (2.49 and 2.97 g/100 g muscle, respectively) than the other genotypes and metabolisms oriented to accumulate dietary resources in the body, according to resource allocation theory and previous results [52]. It is probable that, part of n-3 PUFA ingested by SG with lower DWG, was used for the maintenance of body energy (immune status, thermotolerance, etc) [52] or to supply the energy of these very active chickens thought the β-oxidation pathway [53] (as stated by resource allocation theory).

Therefore, these animals have higher efficiency in converting such dietary nutrients into meat. In foraging birds, part of the energetic requirement for walking activity may be due to β-oxidation of fatty acids [12,52], thus increasing PUFA mobilization and reducing the fat content of chickens [54].

Regardless, whether the higher efficiency of storage was a result of the specific metabolism of the strain or depended on the amount ingested remains unclear. Although the present data do not permit a definitive answer, a comparison of the trends of both PUFA series and antioxidants indicates that an interaction between these factors is probable. Indeed, it is well know that higher PUFA content in meat, if not appropriately balanced with antioxidants compounds (i.e. vitamin E, C, polyphenols, etc) increases the tissue susceptibility to oxidation [55], probably the interaction of pro and anti- oxidative molecules [56], also affected the estimated content of each compounds in chickens meat.

Even if the n-3 LC-PUFA did not show a clear trend (Table 7), it should be emphasized that grass intake of walking chickens is a “natural” way to increase the n-3 LC-PUFA content of meat and magnify the low ALA content of the standard feed. Because outdoor runs are mandatory in ERSs, pasture intake is a plus that can highlight the adaptation of chicken strains. Accordingly, if pasture is considered an extra and free supplementation source, the grass conversion in LC-PUFA was much higher in foraging chickens that that of the other genotypes (0.47 in SG1, 0.38 in SG2, 0.41 in SG3 vs. < 0.30 in all other genotypes; resulted from data reported in Table 7).

The storage efficiency of tocols and carotenes showed a trend similar to that of n-3 PUFA but had higher variability, probably due to different physiological mechanisms (oxidative thrust, kinetic activity, energy balance, etc). In particular, SG1 and SG2 had a lower antioxidant uptake, which means that these genotypes took a high amount of tocols but stored much less quantity, probably due to the involvement of such compounds in the oxidative/antioxidant balance [8,57]. Indeed, more “active” animals have a higher oxidative thrust associated with this activity [8]. This overproduction of free radicals could compromise the antioxidant defence of organism, although neutralization by appropriate levels of antioxidant intake results in a good oxidative status of meat [7,55]. Thus, the more active strains probably had a higher need for supplemental vitamins to counteract radical production.

## Conclusion

Pasture availability is an essential aspect of ERS although it is clear that grass intake does not ensure adequate energy and protein intake by birds. Many aspects may modulate the foraging activity of chickens. The data presented herein suggest that foraging is relevant for nutrient intake because it provides a high share of nutrients, i.e., carotenes, tocols and n-3 PUFA, which are often scarce in standard poultry feed. Accordingly, the foraging chicken genotypes had better meat nutritional profiles (less fat, more n-3 PUFA and LC-PUFA content) than not-foraging ones, due to the intake of grass which is a free-available source of nutrients in ERS.

However, associated with differences in foraging behavior, the genotypes had a negative correlation between the ability to store nutrients provided by feed and/or grass in body meat and their foraging activity. Genetic selection should be used to favor chickens with a good balance between foraging and recovery rates, given that active animals have lower storage efficiencies.

## Supporting information

S1 Table Parameter items recorded in the experimentation. (DOC)
