# Supplementary material for: Intake of nutrients (polyunsaturated fatty acids, tocols, and carotenes) and storage efficiency in different slow-growing chickens genotypes reared in extensive systems
Source: PLoS One. 2022 Nov 1;17(11):e0275527. doi: 10.1371/journal.pone.0275527 (PMC9624413; doi:10.1371/journal.pone.0275527)
Supplement: S1 Table — (DOC) [file pone.0275527.s001.doc]

**Supplementary table S1. Parameter items recorded in the experimentation**

| *Traits* | *Ontology name* | *ATOL ID^1^* | *Description of measurement* |
| --- | --- | --- | --- |
| ADW | average daily gain | ATOL_0000989 | any measurable characteristic related to the change in body weight over a period of time, divided by the number of days in the period |
| walking | walking behaviours | ATOL_0000805 | any measurable or observable characteristic related to coordinated movements to move on foot |
| Feed intake | average daily feed intake | ATOL_0005508 | any measurable characteristic related to the amount of feed consumed daily averaged on a given time period |
| Feed efficiency | animal feed efficiency | ATOL_0002159 | Any measurable or observable characteristic related to the ratio between production output and feed input |
| Muscle yield | muscle yield | ATOL_0000011 | any measurable or observable characteristic related to muscle weight obtained after the processing of a carcass, normalised by the weight of another body part which includes the weighted muscle |
| Feed Ash | feed mineral apparent digestibility | ATOL_0001251 | any measurable or observable characteristic related to the apparent digestibility of feed inorganic substance |
| Feed protein | feed nitrogenous component apparent digestibility | ATOL_0001244 | any measurable or observable characteristic related to the apparent digestibility of feed components able to supply nitrogen |
| Feed lipid | feed lipid apparent digestibility | ATOL_0001249 | any measurable or observable characteristic related to the apparent digestibility of lipid (CHEBI:18059) of feed |
| Fibre | feed crude fibre apparent digestibility | ATOL_0001243 | any measurable or observable characteristic related to the apparent digestibility of the cellulosic fraction of the vegetal parietal components in digesta |
| NDF | feed neutral detergent fibre apparent digestibility | ATOL_0001240 | any measurable or observable characteristic related to the apparent digestibility of feed insoluble residues in the digestive tract |
| ADF | feed acid detergent fibre apparent digestibility | ATOL_0001242 | any measurable or observable characteristic related to the apparent digestibility of the ADF fraction of vegetal parietal components in feed |
| ADL | feed acid detergent lignin apparent digestibility | ATOL_0001241 | any measurable or observable characteristic related to the apparent digestibility of the lignocellulosic fraction of the vegetal parietal components in digesta |
| Feed vitamin A and E | feed vitamin apparent digestibility | ATOL_0001255 | any measurable or observable characteristic related to the apparent digestibility of vitamin (CHEBI:50211) of feed |
| Meat moisture | meat moisture level | ATOL_0001668 | any measurable characteristics of the amount of moisture in a cut of meat, determined by the difference in weight before and after oven drying |
| Meat ash | meat mineral and ash content | ATOL_0001695 | amount of inorganic residue remaining after the removal of water and organic material. A measure of the total mineral content |
| Meat fat | meat lipid content | ATOL_0001663 | any measurable or observable characteristic related to the amount of fat-soluble substances in meat (molecules composed of carbon and hydrogen characteristically insoluble in water, lipid: CHEBI:18059) |
| Meat vitamin | vitamin content of meat | ATOL_0000062 | any measure of the quantity of the different vitamins (CHEBI:33229) in meat |
| Meat fatty acids profile | meat fatty acid content | ATOL_0000058 | Any measure of the quantity of different fatty acids (CHEBI:35366) present in the meat |
| Foraging | grazing behaviour | ATOL_0001780 | any measurable or observable characteristic related to eat on herbage, algae or peryphyton |
| Grass | physical environment investigation | ATOL_0000845 | any measurable or observable characteristic related to the investigation of the physical environment through sensorial paths such as sniffing, pecking, scratching, biting, looking at. |

^1^ Traits adapted from references to the ontology ATOL: <http://www.atol-ontology.com/index.php/en/>
